# Patient journey for hypertension and dyslipidemia in Saudi Arabia: highlighting the evidence gaps

**DOI:** 10.1186/s13690-023-01121-3

**Published:** 2023-07-03

**Authors:** Ashraf Amir, Mirvat Alasnag, Rajaa Al-Raddadi, Tawfik Al-Bassam, Kanwal Saeed, Mehmet Yazıcıoğlu, Ayman Shabana

**Affiliations:** 1 Family Medicine Department, International Medical Center, Jeddah, Saudi Arabia; 2 Cardiac Center, King Fahd Armed Forces Hospital-Jeddah-Saudi Arabia, PO Box 9862, Jeddah, 21159 Saudi Arabia; 3 Faculty of Medicine, Department of Community Medicine, King Abdulaziz University, Jeddah, Saudi Arabia; 4 Department of Internal Medicine, Medical Reference Center, Jeddah, Saudi Arabia; 5 Research, Development and Medical, Pfizer Upjohn, Dubai, UAE; 6 Emerging Markets Medical Portfolio Implementation Lead, Viatris, Istanbul, Turkey; 7 Viatris, Jeddah, Saudi Arabia

**Keywords:** Cardiovascular disease, Dyslipidemia, Hypertension, Patient journey, Prevalence, Kingdom of Saudi Arabia

## Abstract

**Background:**

In recent years, Saudi Arabia has witnessed staggering rates of hypertension and dyslipidemia-related cardiovascular (CV) deaths, overburdening the healthcare ecosystem of the country. Appropriate public health interventions can be devised through quantitative mapping of evidence. Identification of potential data gaps can prioritize future research needs and develop a ‘best-fit’ framework for patient-centric management of hypertension and dyslipidemia.

**Methods:**

This review quantified data gaps in the prevalence and key epidemiological touchpoints of the patient journey including awareness, screening, diagnosis, treatment, adherence, and control in patients with hypertension and dyslipidemia in Saudi Arabia. Studies published in English between January 2010 and December 2021 were identified through a structured search on MEDLINE, Embase, BIOSIS, and PubMed databases. An unstructured search on public and government websites, including Saudi Ministry of Health, without date limits was carried out to fill data gaps. After exclusion of studies based on predefined criteria, a total of 14 studies on hypertension and 12 studies and one anecdotal evidence for dyslipidemia were included in the final analyses.

**Results:**

The prevalence of hypertension was reported to be 14.0%–41.8% while that for dyslipidemia was 12.5%–62.0%. The screening rate for hypertension was 100.0% as revealed by the nationwide surveys. Among hypertensive patients, only 27.6%–61.1% patients were aware of their condition, 42.2% patients underwent diagnosis, 27.9%–78.9% patients received antihypertensive treatment, 22.5% patients adhered to treatment medication, while blood pressure (BP) control was achieved in 27.0%–45.0% patients. Likewise, among patients with dyslipidemia, 10.5%–47.3% patients were aware of their condition, 34.6% patients were screened, and 17.8% underwent diagnosis. Although high treatment rates ranging from 40.0%–94.0% were reported, medication adherence recorded was 45.0%–77.4% among the treated patients. The overall low control rates ranged from 28.0%–41.5%.

**Conclusions:**

The study findings highlight evidence gaps along key touchpoints of patient journey. Reinforcing the efforts for high-quality evidence-based research at a national level may pave a path for better resource utilization and provide guidance to practice and amend health policies for patients, healthcare practitioners (HCPs), and healthcare policy makers for better patient outcomes in Saudi Arabia.

**Supplementary Information:**

The online version contains supplementary material available at 10.1186/s13690-023-01121-3.

## Background

The burden of noncommunicable diseases (NCDs) has been a growing challenge faced by the global community. The mortality due to NCDs has undergone a huge transition particularly across Western countries and extending to countries in the Middle East North Africa (MENA) region and Asia [1, 2]. Saudi Arabia has been at the cusp of this shift with a reported 73.0% of all deaths attributed to NCDs [2]. The ever-increasing population of Saudi Arabia along with major lifestyle changes, rapid economic and technological growth has exposed the country to a myriad of lifestyle-related NCDs including hypertension, dyslipidemia, diabetes mellitus (DM), obesity, and coronary artery diseases (CAD) [3]. Likewise, the exponential increase in life expectancy (45.6–74.9 years) by nearly 30.0 years over the past six decades has also led to a corresponding rise in NCDs [4]. Although the mean age of overall population remains low compared with Western countries, recent estimates suggest incremental increase in the aging population from 5.6% in 2017 to 22.9% in 2050 [5]. Cardiovascular diseases (CVDs) remain a major cause of deaths (37.0%) in Saudi Arabia [6]. Although hypertension, smoking, DM, and dyslipidemia are the major modifiable risk factors for CVDs, emerging robust data suggest hypertension to be an independent risk factor strongly linked with various CVD complications [7, 8]. Likewise, dyslipidemia is associated with a two-fold higher risk of CVD causation [9].

Huge financial investments have been made by the Saudi government in the healthcare sector along with the provision of accessible healthcare services. Despite this, a high prevalence of hypertension (15.2%, hypertension; 40.6%, prehypertension) and dyslipidemia (43.0%) has been reported by two recent nationwide surveys [10, 11]. Understandably, there are certain barriers for patients and healthcare practitioners (HCPs) that can hinder adequate utilization of healthcare facilities. From the patients’ perspective, undermining the importance of blood pressure (BP) screening, inconsistent follow-up with the primary healthcare clinics (PHCCs) due to limited technical knowledge to avail online consultations, variations in the distribution of PHCCs across urban and rural regions, lower number of HCPs in PHCCs (lower by 40% vs. hospitals), are some of the hindrances in the patient journey [12–14]. As for the HCPs, there is a notably low adherence to treatment guidelines and an absence of validated risk assessment tools designed for the local population. This leads to missed screening opportunities in appropriate individuals that can constitute a significant barrier for timely diagnosis and treatment and may lead to poor CVD prognosis [15]. Additionally, socioeconomic factors associated with CVDs, gender-based inequalities, regional differences, and diverse cultural norms across different regions of Saudi Arabia may constitute another hurdle in primary prevention efforts [1, 12].

Frequent nationwide and regional screening campaigns, constant and unbiased medical communication between patients and HCPs, and patient engagement programs in the community settings can lead to better healthcare facilities utilization, thereby reducing the burden of hypertension and dyslipidemia in Saudi Arabia [12]. A longitudinal patient journey map can reflect on the critical needs of managing these NCDs from the patients’ perspective and that can help strategizing the treatment goals, improving the patient-HCP communication, and eventually patient care [16]. This can be achieved by mapping patients’ disease journey through six broad touchpoints, namely awareness, screening, diagnosis, treatment, adherence, and control.

Previously, a national survey that mapped patient journeys in the Saudi population in 2005 reported > 1200 participants to be hypertensive with less than half of them being aware, receiving treatment, and having their BP controlled [17]. However, the studies reporting patient journeys in the following years were region-specific individual studies done at different times [18]. This may not provide an accurate estimate across all the journey touchpoints within the Saudi population who already have varied cultural norms and behavior towards CVD management [19]. Under such circumstances, a literature review with quantitative mapping and data visualization including the patient journey touchpoints can identify patients at-risk by providing a holistic perspective on risk factors stratified by age, gender, and dwelling place (urban or rural). Moreover, no Saudi studies could map the CVD care continuum through the lens of diverse settings, i.e., community or multilevel healthcare. Such data mapping can reflect upon the missed opportunities along the care continuum within both the settings—community healthcare: for risk factor identification and modification; and diagnosis and healthcare: for targeted treatments (first line and advanced) and palliative care [20]. Through this review, the overall evidence gap map thus generated can interest the researchers and clinicians to implement necessary steps towards closing the gap and provide nuanced care towards CVDs while ensuring smooth transition through each level of patient journey.

Traditionally, high-quality research outcomes focus on a few select developed countries, wherein the data across global population is extrapolated to locally design country-specific or region-specific guidelines. This results in inaccurate patient journey mapping due to lack of real-world local data [21]. Localized data mapping can aid global policy makers design priority framework from the viewpoint of recommending nutrition and lifestyle along with estimating the overall economic burden resulting from CVDs. From the standpoint of Saudi Arabia, such evidence mapping studies help the international agencies guide Saudi Arabia towards an optimal utilization and distribution of resources, maintenance of inventories, and development of policies and strategies to help shape up practical approaches towards CVD management in the country [21, 22]. The evidence mapping can also ensure preparedness of policy makers and practitioners in prioritizing at-risk patient pool and budget infrastructure in an event of unprecedented health crisis, e.g., coronavirus disease 2019 (COVID-19) [23]. Furthermore, implementation of health programs and policies along with elaboration of comprehensive reforms and recommendations and devising prioritization matrices for future epidemiological research efforts within the Saudi population can maneuver the policies and improve overall health status of the country. Table 1 highlights a few of the many roles of evidence mapping studies in shaping healthcare policies on a local and global level.

**Table 1:** Roles of evidence mapping studies in shaping healthcare policies on a local and global level

• Providing a holistic perspective on CVD risk factors in a population stratified by age, gender, and dwelling place (urban or rural)
• Circumscribing CVD care continuum across diverse settings (community-based or multilevel healthcare)
• Ensuring targeted and nuanced care through each level of disease journey to improve patient outcomes
• Designing priority framework by global policy makers and act as guiding force for optimal resource utilization and distribution on a national level
• Ensuring preparedness in terms of policies and resource budgeting to tackle unprecedented health crisis based on at-risk patient pool
• Devising prioritization matrices for future epidemiological research efforts

The objective of this review is to present the data across various patient journey touchpoints (prevalence, awareness, screening, diagnosis, treatment, adherence, and control) of hypertension and dyslipidemia in Saudi Arabia to further strengthen the healthcare system and overall quality of care by providing direction. To achieve this objective, the following research questions were designed: How much is the evidence gap along each journey touchpoint specifically in the Saudi population barring the expatriates since last 10 years? What is the reason that led to those gaps?

## Methods

### Review design

An extensive literature review was conducted using structured and unstructured search strategies to identify studies dealing with patient journey touchpoints (awareness, screening, diagnosis, treatment, adherence, and control) for hypertension and dyslipidemia in Saudi Arabia. Hypertension was defined as an average systolic blood pressure (SBP) ≥ 140 mmHg and/or average diastolic blood pressure (DBP) ≥ 90 mmHg. Dyslipidemia was defined as total cholesterol (TC) of ≥ 5.0 mmol/L or ≥ 200.0 mg/dL [21]. Definitions of each patient journey touchpoint are provided in Additional file 1. The Preferred Reporting Items for Systematic Reviews and Meta-Analyses (PRISMA) guidelines were used in this review, with minor modifications to fit within the scope of this study. The evidence was mapped using MAPS (Mapping the Patient Journey Towards Actionable Beyond the Pill Solutions) methodological approach [18, 21]. The mapping strategy was divided into six steps: (1) developing a comprehensive search strategy; (2) establishing the inclusion and exclusion criteria; (3) screening and shortlisting; (4) supplementing with additional and/or local data; (5) data extraction and synthesis; and (6) evidence mapping.

### Search strategy

An electronic structured search was conducted to identify studies published from 01 January 2010 to 31 December 2021 through Medline and Embase databases using the keywords related to hypertension and dyslipidemia and different touchpoints of patient management. The comprehensive search was designed to ensure the inclusion of all studies conducted in Saudi Arabia on hypertension and dyslipidemia. The complete search strategy is provided in Additional file 2.

To address data gaps in the structured search, an additional unstructured search was conducted through the Incidence and Prevalence Database (IPD), World Health Organization (WHO), Country’s Ministry of Health (MOH), and Google Scholar websites. No date limits were applied to the unstructured search. Data gaps were further supplemented with anecdotal data from clinical experts in Saudi Arabia.

### Eligibility criteria

Eligible publications were based on: (a) studies on the human adult population (aged ≥ 18 years) with hypertension and dyslipidemia that focused on the prevalence and epidemiological data from different patient journey touchpoints (awareness, screening, diagnosis, treatment, adherence, and control or remission); (b) peer–reviewed published systematic review and/or meta-analysis, narrative reviews, observational studies; (c) studies representing patient population from Saudi Arabia.

Studies with special populations such as pregnant women; patients with comorbidities, non-English language studies, thesis abstracts; letters to the editor, and editorials; studies including specific patient subgroups; duplicate records and studies without full text were excluded from the analysis.

### Screening of studies

The first independent reviewer retrieved the studies using both structured and unstructured search strategies. Studies were screened and selected based on their title and abstract. The second independent reviewer assessed the selected studies considering predetermined inclusion/exclusion criteria and reviewed the full text. Any disagreements between the first and the second reviewer were resolved to arrive at a mutual consensus. A comprehensive review through manual screening of all the selected studies was carried out again, prior to data extraction and synthesis. Furthermore, any identified data gaps were supplemented with anecdotal data from clinical experts in Saudi Arabia.

### Data extraction and synthesis

Following a thorough manual screening of all the selected studies, relevant studies were shortlisted and exported to the data extraction grid in Microsoft Excel. Quantitative data pertaining to the various patient journey touchpoints (awareness, screening, diagnosis, treatment, adherence, and control) were captured in the grid. The data extraction grid was rechecked and verified by the reviewers for consistency and accuracy. The grid was further synthesized to highlight the evidence gap map for both hypertension and dyslipidemia.

## Results

### Screening of studies for hypertension

**Fig. 1:**
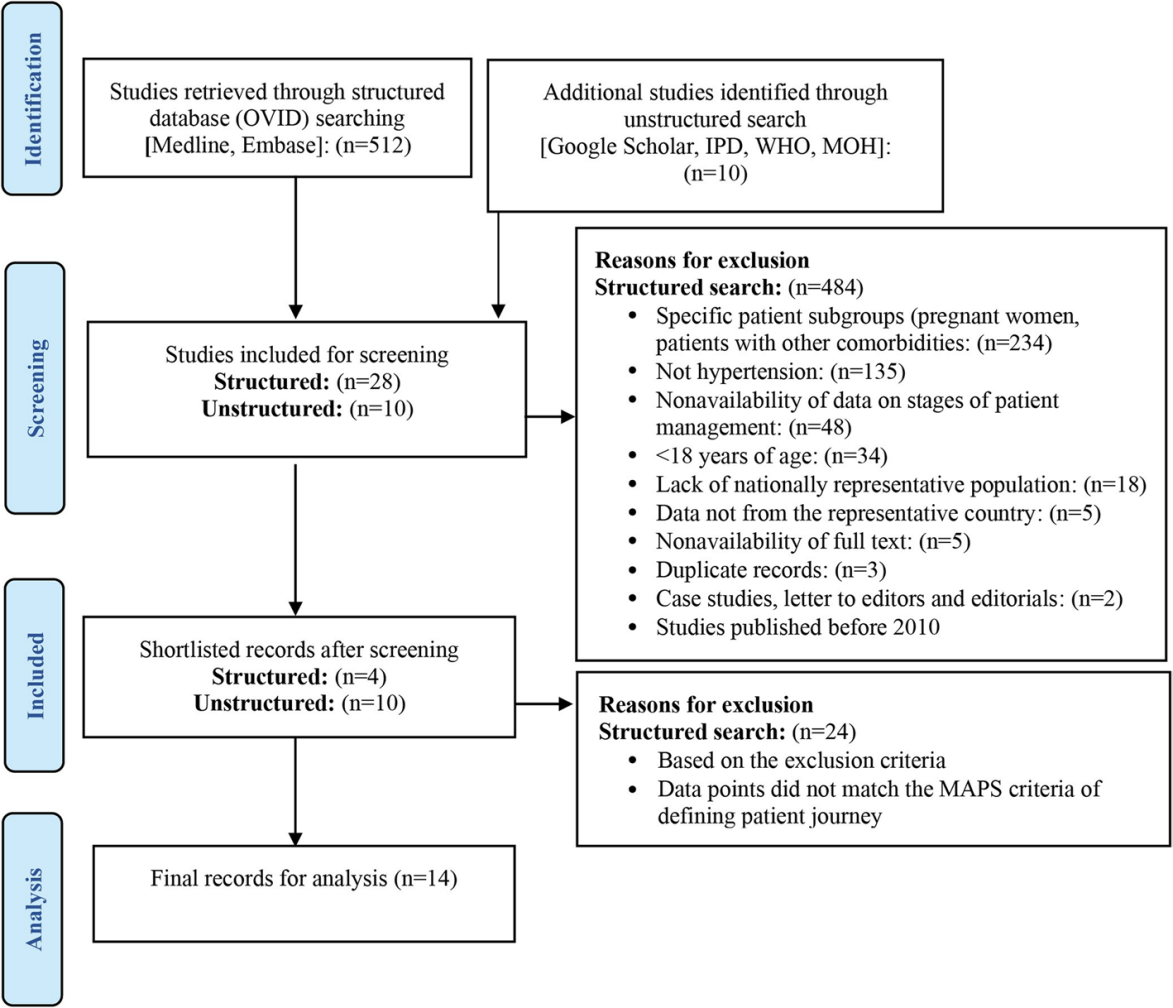
Flowchart describing the inclusion of hypertension-related studies in the final analysis. Abbreviations: IPD, Incidence and Prevalence Database; MAPS, Mapping the Patient Journey Towards Actionable Beyond the Pill Solutions; MOH, Ministry of Health; n, number of studies; WHO, World Health Organization

A total of 524 studies were retrieved from the structured and unstructured searches on the prevalence and the patient journey touchpoints of hypertension. Of these, 512 studies were obtained through structured search and 12 studies from unstructured search. Most of the excluded studies represented specific patient subgroups (pregnant women, patients with other comorbidities, *n* = 234) and diseases other than hypertension (*n* = 135). Other reasons for exclusion of studies were nonavailability of data on patient journey touchpoints (*n* = 48),  < 18 years of age (*n* = 34), lack of nationally representative population (*n* = 18), data not from Saudi Arabia (*n* = 5), nonavailability of full text (*n* = 5), duplicate studies (*n* = 4), case studies (*n* = 2), studies published before 2010 (*n* = 1). Twenty-eight studies from the structured and 10 studies from the unstructured searches were selected for detailed review. Twenty-four studies from the structured search were excluded as they did not match the MAPS criteria definition for patient journey touchpoints. Finally, four studies from the structured searches [10, 17, 24, 25] and 10 studies from the unstructured searches [11, 26–34] were included in the final analyses. The literature search and study selection process are presented in Fig. 1.

### Description of included studies for hypertension

The included studies were conducted both in community-based and hospital-based settings. The overall prevalence of hypertension in community-based settings ranged from 14.0%–36.0% [10, 11, 17, 25, 26, 29, 34], while the prevalence in hospital-based settings ranged from 14.8%–41.8% [24, 30, 31]. The prevalence data as reported in two studies, i.e., Global Status report on NCDs and the Kingdom of Saudi Arabia World Health Survey (KSA WHS), both in collaboration with WHO, ranged from 14.0%–21.8% [11, 26]. A nationwide multistage random sampling carried out in 20 health regions of Saudi Arabia reported a hypertension prevalence of 25.5%; 44.7% patients were aware of their condition, 71.8% patients received treatment for their condition, and 37.0% patients achieved hypertension control [17]. A subgroup analysis of the Africa Middle East Cardiovascular Epidemiological (ACE) study revealed a prevalence of 41.8% among young Saudi patients and expatriates [17].

A nationwide multistage survey carried out by El Bcheraoui et al. reported an overall prevalence of hypertension of 15.2%; further, 42.2% patients were diagnosed with hypertension [10]. About 78.9% of the previously diagnosed hypertensive patients were receiving treatment for hypertension management and 45.0% of the treated patients had achieved control [10]. Two studies involving Saudi national subgroup analysis of the Prospective Urban Rural Epidemiology (PURE) study reported data that were both standardized and non-standardized by age. The first subgroup analysis reported hypertension prevalence to be 36.0%; 56.0% patients were aware of their condition, 53.0% patients were receiving treatment, and 27.0% patients were having controlled BP [25]. Similarly, the second subgroup analysis reported hypertension prevalence (non-standardized by age) to be 30.3%; further, 61.1% patients were aware of their condition, 58.9% patients received treatment, and 30.7% reported hypertension control [29].

A nationwide screening campaign as a part of the global screening initiative, May Measurement Month, reported 100.0% screening of hypertension [26]. The campaign reported that 29.2% patients were hypertensive, 60.8% patients were aware of their condition and were receiving treatment, and 39.3% patients had their BP controlled [30]. One university-based study revealed that hypertension was prevalent among 31.0% patients [34], while one study reported 27.6% of patients to be aware of their condition [32]. The screening rates among the patients was reported to be 100.0% from three studies [30, 32, 34]. As per the data obtained from two cross-sectional studies, the average prevalence of hypertension was found to be 14.8% [29], whereas the awareness, stratified by the patients’ knowledge of SBP and DBP was reported to be 48.7% and 47.3%, respectively [33]. The detailed study characteristics are presented in Table 2.

**Table 2:** Overview of the hypertension related studies included in the final analysis

Sr No	Study: first author; publication date	Brief study design	Sample size (N); characteristics	Prevalence (%)	Awareness (%)	Screening (%)	Diagnosis (%)	Treatment (%)	Adherence (%)	Control (%)
**Structured search**										
1	Prevalence, awareness, treatment, and control of hypertension among Saudi adult population: A national survey: Saeed A.A et al.; 2011 [17]	Cross–sectional community–based study	*N* = 4,758 enrolled from 20 health regions of Saudi Arabia; aged 15.0–64.0 years	25.5	44.7	X	X	71.8	X	37.0
2	Cardiovascular risk factors burden in Saudi Arabia: The Africa Middle East Cardiovascular Epidemiological (ACE) study Ahmed A.M et al.; 2017 [24]	Cross–sectional epidemiological study (subgroup analysis)	*N* = 550 enrolled from different clinics across Saudi Arabia; mean age 43.2 ± 10.5 years	41.8	X	X	X	X	X	X
3	Hypertension and its associated risk factors in the Kingdom of Saudi Arabia, 2013: A national survey: El Bcheraoui C et al.; 2014 [10]	National multistage survey	*N* = 10,735 enrolled from 13 regions of Saudi Arabia; aged 15.0– > 65.0 years	15.2	X	X	42.2	78.9	X	45.0
4	Prevalence, awareness, treatment, and control of hypertension in four Middle East countries: Yusufali A.M et al.; 2017 [25]	Large-scale epidemiological study (PURE study)	*N* = 1,56,424 enrolled from 628 communities in 17 countries; aged 35.0–70.0 years *n* = 2041 enrolled from Saudi Arabia; mean age 48.0 ± 9.4 years, (M); 45.0 ± 8.5 years (F)	36.0	56.0	X	X	53.0	X	27.0
**Unstructured search**										
5	KSA WHS 2019: Ministry of Health; 2021 [11]	Population–based survey	*N* = 8,912 enrolled from 13 administrative regions; aged ≥ 15.0 years	14.0	X	X	X	X	X	X
6	Global Status report on noncommunicable diseases 2014 [26]	Global status report tracking worldwide progress in prevention and control of NCDs	Adults (> 18.0 years)	21.8	X	X	X	X	X	X
7	Factors affecting antihypertensive medications adherence among hypertensive patients in Saudi Arabia: Alsolami F et al.; 2015 [27]	Cross–sectional study	*N* = 308 enrolled from outpatient department of the hospital; aged ≥ 18.0 years	X	X	X	X	27.9	X	X
8	Predictors of medication adherence and blood pressure control among Saudi hypertensive patients attending primary care clinics: A cross-sectional study: Khayyat S.M. et al.; 2017 [28]	Prospective cross–sectional study	*N* = 204 enrolled from eight different PHCs; aged > 18.0 years	X	X	X	X	X	22.5	X
9	Demographic, behavioral, and cardiovascular disease risk factors in the Saudi population: results from the Prospective Urban Rural Epidemiology study (PURE-Saudi): Alhabib K.F. et al.; 2020 [29]	Cohort study (subgroup analysis)	*N* = 2,047 enrolled from 19 urban and six rural communities; mean age 46.5 ± 9.1 years	30.3	61.1 (of 30.3)	X	X	58.9	X	30.7
10	May measurement month 2019: an analysis of blood pressure screening results from Saudi Arabia: Aljuraiban G.S. et al.; 2021 [30]	Awareness initiative in collaboration with the Saudi Ministry of Health	*N* = 25,023 enrolled from 92 primary care centers aged ≥ 18.0 years	29.2	60.8	100.0	X	60.8	X	39.3
11	The association between hypertension and other cardiovascular risk factors among nondiabetics Saudis adults–A cross sectional study: Ajabnoor G.M.A. et al.; 2021 [31]	Cross sectional study	*N* = 1334 enrolled from PHCs	14.8^a^	X	X	X	X	X	X
12	Social knowledge of symptoms, risk factors, causes and complications of hypertension among Al-Ahsa population, Saudi Arabia: Elsheikh E et al.; 2021 [32]	Cross-sectional study	*N* = 660 enrolled from multiple PHCs; aged ≥ 18.0 years	X	27.6	100.0	X	X	X	X
13	Are patients affected by chronic non-communicable diseases aware of their own clinical and laboratory parameters? A cross-sectional study from the south of Saudi Arabia: Gosadi I.M.et al.; 2021 [33]	Cross-sectional study	*N* = 675 enrolled from 65 PHCs; aged 18.0–95.0 years	X	48.7 (SBP) 47.3 (DBP)	X	X	X	X	X
14	Prevalence of diabetes and hypertension among King Abdulaziz University Employees: Data from first aid and cardiopulmonary resuscitation training program: Khafaji M.A. et al.; 2021 [34]	Retrospective study	*N* = 1000 enrolled from the university campus; aged 21.0– > 60.0 years	31.0	X	100.0	X	X	X	X

*Abbreviations: ACE* The Africa Middle East Cardiovascular Epidemiological study, *F* females, *KSAWHS* Kingdom of Saudi Arabia World Health Survey, *M* males, *N* total sample size; n, sub-set sample size, *NCDs* non-communicable diseases, *PURE* Prospective Urban Rural Epidemiology, *PHCs* primary healthcare, *SHIS* Saudi Health Interview Survey

^a^Average value calculated for males and females

### Screening of studies for dyslipidemia

**Fig. 2:**
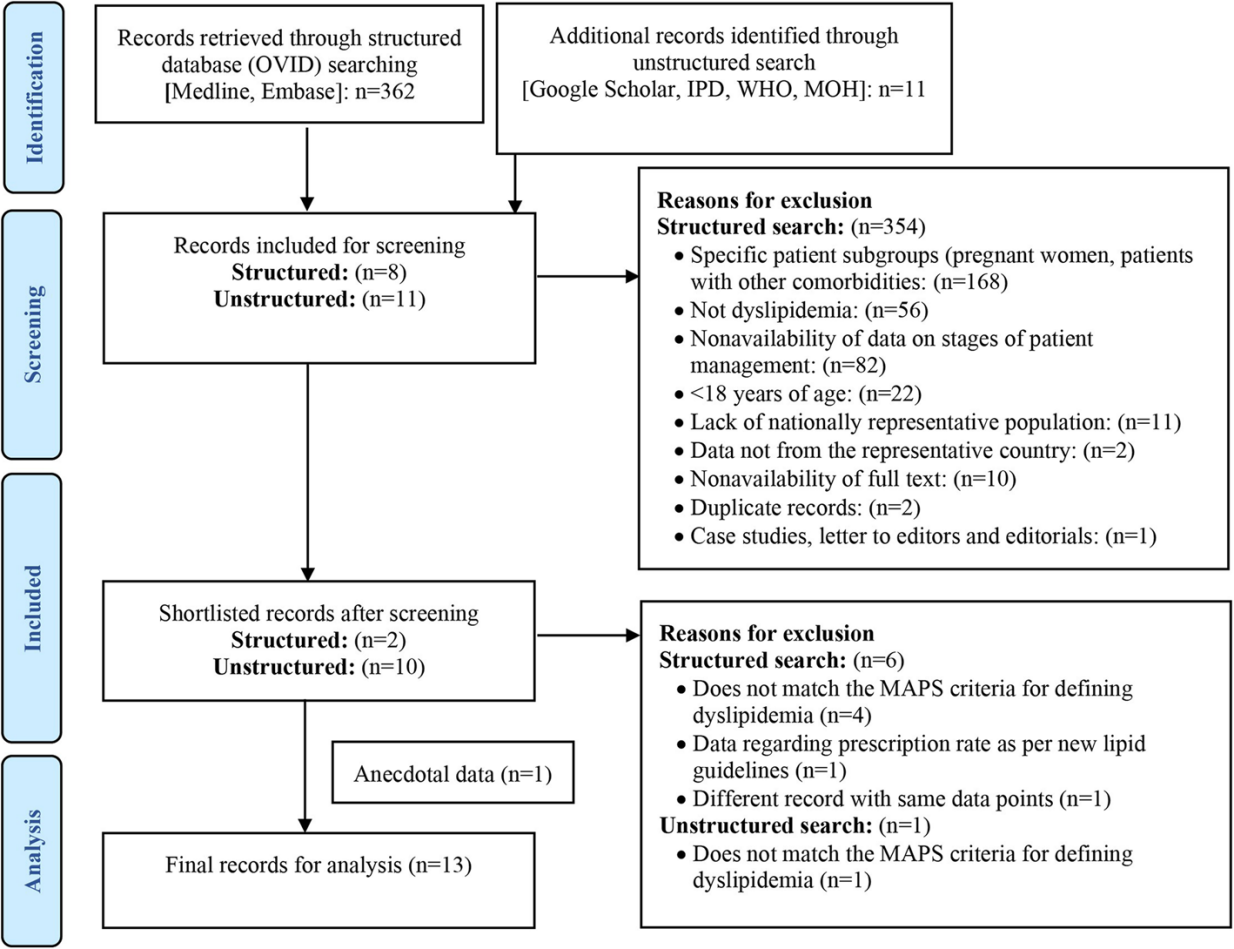
Flowchart describing the inclusion of dyslipidemia-related studies in the final analysis. Abbreviations: IPD, Incidence and Prevalence Database; MAPS, Mapping the Patient Journey Towards Actionable Beyond the Pill Solutions; MOH, Ministry of Health; n, number of studies; WHO, World Health Organization

A total of 373 studies were retrieved from the structured and unstructured searches on the prevalence and the patient journey stages of dyslipidemia. Of these, 362 studies were found through structured search and 11 studies through unstructured search. Most of the excluded studies represented specific patient subgroups (pregnant women, patients with other comorbidities, *n* = 168) and did not have dyslipidemia (*n* = 56). Other reasons for exclusion of studies were non-availability of data on stages of patient journey touchpoints (*n* = 82),  < 18 years of age (*n* = 22), lack of nationally representative population (*n* = 11), data not from Saudi Arabia (*n* = 2), non-availability of full text (*n* = 10), duplicate records (*n* = 2), and editorials (*n* = 1). Finally, two studies from the structured searches [35, 36] and 10 studies from the unstructured searches [11, 29, 33, 37–43] were included. To fill the data gap, one anecdotal evidence was included in the final analyses. The literature search and study selection process are presented in Fig. 2.

### Description of included studies for dyslipidemia

The studies from community-based settings reported a prevalence ranging from 12.5%–43.0% [11, 29, 43], while the prevalence in hospital-based settings was 62.0% [41]. As per the data reported by KSA WHS, dyslipidemia was prevalent in 43.0% of the Saudi population [11].

The Saudi national subgroup analysis of the PURE study reported the prevalence to be 32.1% [29]. Two cross-sectional survey studies reported the dyslipidemia prevalence to be 12.5% and 62.0%, respectively [41, 43]. A Saudi national subgroup analysis of the SHIS (Saudi Health Interview Survey) study revealed that 17.8% participants who self-rated their health to be poor/fair were diagnosed with dyslipidemia [35]. Lipid profile screening rate was reported in only one cross-sectional survey study (34.6%) [38]. A multinational survey study, which included Saudi Arabia, reported a very high proportion of patients (94.0%) undergoing treatment, particularly with statins [36]. Likewise, the Saudi national subgroup analysis of PURE study indicated that 40.6% of the patients were undergoing lipid-lowering treatment as secondary prevention of CVD [39]. Similarly, in a prospective cohort study, although 40.0% reported as being prescribed the lipid-lowering drugs, only 15.0% received appropriate dosing [42].

The Morisky Medication Adherence Scale (MMAS) assessing medication adherence rate reported that 26.0% patients adhered to the therapy which increased to 62.0% over nine months [42]. A retrospective cross-sectional study that captured data from electronic health records (EHRs) of patients with type 2 diabetes (T2D) and dyslipidemia showed 77.4% adherence to lipid-lowering treatment, while 41.5% of the patients displayed control [40]. According to the anecdotal data provided by Dr. Ashraf Amir, 45.0% patients were found to be adherent to the treatment. The study details are summarized in Table 3.

**Table 3:** Overview of the dyslipidemia related studies included in the final analysis

Sr No	Study: first author; publication date	Brief study design	Sample size (N); characteristics	Prevalence (%)	Awareness (%)	Screening (%)	Diagnosis (%)	Treatment (%)	Adherence (%)	Control (%)
**Structured search**										
1	Self-Rated Health Among Saudi Adults: Findings from a National Survey, 2013: Moradi-Lakeh M; 2015 [35]	National multistage survey	*N* = 10,735 enrolled from 13 regions of Saudi Arabia; aged ≥ 15.0 years	X	X	X	17.8	X	X	X
2	Control of Risk Factors for Cardiovascular Disease among Multinational Patient Population in the Arabian Gulf: Al-Zakwani I; 2016 [36]	Multi-center non-interventional survey	*N* = 3,259 enrolled from outpatient clinics; aged ≥ 18 years; mean age 56.0 ± 11.0 years	X	X	X	X	94.0	X	28.0
**Unstructured search**										
3	Level of awareness regarding hypercholesterolemia, Saudi Arabia, Riyadh, 2017: Al-Qahtani M et al.; 2017 [37]	Cross-sectional study	*N* = 150 enrolled from Saudi Arabia; age ≥ 18.0 years)	X	47.3^a^/ 28.6^b^	X	X	X	X	X
4	Awareness among the general population about lipid profile screening in individuals over 20 years old in Alriyadh, Saudi Arabia: Bahakim NO; 2019 [38]	Cross–sectional survey study	*N* = 1,383 surveyed through a self-constructed electronic questionnaire; aged > 20.0 years	X	X	34.6	X	X	X	X
5	Inequalities in the use of secondary prevention of cardiovascular disease by socioeconomic status: evidence from the PURE observational study: Murphy A et al.; 2018 [39]	A large observational international study	*N* = 2,047 enrolled from urban and rural communities in Saudi Arabia; mean age, 46·5 ± 9·1 years	X	X	X	X	40.6	X	X
6	Adherence to Statin Therapy and Attainment of LDL Cholesterol Goal Among Patients with Type 2 Diabetes and Dyslipidemia: Alwhaibi M et al.; 2019 [40]	Retrospective, cross-sectional EHRs review study	*N* = 1,397 enrolled from outpatient clinics at a university–affiliated tertiary care center; aged ≥ 18.0 years	X	X	X	X	X	77.4	41.5
7	Demographic, behavioral, and cardiovascular disease risk factors in the Saudi population: results from the Prospective Urban Rural Epidemiology study (PURE-Saudi): Alhabib K.F; 2020 [29]	Large-scale epidemiological study (PURE study)	*N* = 2,047 enrolled from 19 urban and six rural communities; mean age 46.5 ± 9.1 years	32.1	X	X	X	X	X	X
8	The Association between Dyslipidemia, Dietary Habits and Other Lifestyle Indicators among Non-Diabetic Attendees of Primary Health Care Centers in Jeddah, Saudi Arabia: Enani S et al.; 2020 [41]	Cross–sectional survey	*N* = 1,477 enrolled from PHCs in Saudi Arabia, aged ≥ 20.0 years	62.0	X	X	X	X	X	X
9	Effect of Multidisciplinary Dyslipidemia Educational Program on Adherence to Guidelines Directed Medical Therapy in Saudi Arabia; AlAyoubi F et al.; 2021 [42]	Prospective cohort study	*N* = 401 enrolled from PCC, cardiology clinic, and endocrinology clinic; mean age 60.0 ± 13.0 years; 62.0% (M)	X	X	X	X	40.0^c^/15.0^d^	26.0^e^/62.0	X
10	The prevalence of hypercholesterolemia and associated risk factors in Al-Kharj population, Saudi Arabia: a cross-sectional survey; Al-Zahrani J et al.; 2021 [43]	Cross–sectional study	*N* = 1,019 enrolled from the general population of Al-Kharj, Saudi Arabia; aged ≥ 18.0 years	12.5	X	X	X	X	X	X
11	KSA WHS 2019, Ministry of Health, 2021 [11]	Population–based survey	*N* = 8,912 enrolled from 13 administrative regions; aged > 15 years	43.0	X	X	X	X	X	X
12	Are patients affected by chronic non-communicable diseases aware of their own clinical and laboratory parameters? A cross-sectional study from the south of Saudi Arabia: Gosadi I.M. et al.; 2021 [33]	Cross–sectional study	*N* = 675 enrolled from 65 PHCs; aged 18.0–95.0 years; mean age, 53.7 ± 13.4 years	X	10.5	X	X	X	X	X
**Anecdotal data**										
13	Dr. Ashraf Abdul Qayoum Amir			X	X	X	X	X	45.0	X

*Abbreviations: EHRs* Electronic Health Records, *F* females, *KSAWHS* Kingdom of Saudi Arabia World Health Survey, *LDL* Low-density Lipoprotein, *M* males, *N* total sample size, *PCC* Primary Healthcare, *PHCCs* Primary Healthcare Clinics, *PURE* Prospective Urban Rural Epidemiology, *SHIS* Saudi Health Interview Survey

^a^ Denotes good awareness

^b^Denotes moderate awareness

^c^Number of patients receiving lipid lowering treatment

^d^Number of patients receiving appropriate dosing

^e^Percent patients aware of their dyslipidemia before educational intervention

## Discussion

This review presents the data across the patient journey touchpoints (awareness, screening, diagnosis, treatment, adherence, and control) for hypertension and dyslipidemia. However, there are inconsistencies across the number of studies included for each touchpoint leading to evidence gaps for both hypertension and dyslipidemia. By highlighting the gaps, the HCPs and healthcare policymakers of Saudi Arabia can be encouraged to design more research programs and develop more publications, which in turn will aid in evidence-based informed decision-making in routine clinical practice.

### Hypertension: Gaps along patient journey touchpoints

The overall prevalence estimates of hypertension were represented in a wide range, which could be attributed to the heterogeneity of patients’ baseline characteristics, differences in the study settings (community-based or hospital-based), and difference in the severity of the disease. The prevalence reported from community-based settings were lower (14.0%–36.0%) [10, 11, 17, 25, 26, 29, 34, 35] than in hospital-based settings (14.6%–41.8%) [24, 30, 31], which is indicative that a majority of patients visiting the PHCC were with underlying cardiovascular (CV) risk factors, hypertension being one of them. This was in agreement with the results from previous studies that also showed higher prevalence of hypertension (30.0%–92.0%) [44, 45] in hospital settings compared with community-based studies (13.6%–26.1%) [46, 47]. Additionally, other reasons may include differences in the study duration between the two settings and its corresponding effect on disease severity and differences in diagnostic practices across different locations [24].

Majority of the studies revealed hypertension awareness to be strikingly low in male patients, rural population, and younger patients [25, 29, 30, 32]. On the contrary, increased awareness was observed among female patients, urban dwellers, geriatric patients, and those with chronic comorbidities [25, 29]. These findings were consistent with previous reports wherein heightened awareness was observed among females vs. males [48], urban population, and in patients with DM and CVD. [25] Although Saudi Arabia displays heightened hypertension awareness when compared with other Middle East countries, the hypertension awareness rates were still lower in comparison to other high-income countries (HICs) such as Germany and South Korea (40.0%–80.0%) [25, 49, 50]. Except for two screening campaigns held nationwide and in Eastern province, respectively, the review findings suggested a paucity in screening studies across Saudi Arabia [31, 32, 34]. The reason for high screening rates found in these studies (100.0%) is simply because all the patients underwent screening as part of the survey protocol which did not include targeted screening. However, previously reported community-based screening campaigns taking place in Eastern Province reported lower participant turnout (ranging from 21.0%-33.0%) [49, 50] for screening owing to refusal of undergoing long tedious screening procedures or lack of incentive schemes for participation [51]. Overall, the screening rates remained low in Saudi Arabia which was also similar to that observed in other Middle Eastern countries including United Arab Emirates (UAE), Iran, and Occupied Palestinian Territory (OPT) [25].

Low diagnosis rates (47.1%) trace back to lower rates of awareness and screening campaigns in Saudi Arabia. Although rates of diagnosis have improved over the last decade (from 11.5% to 47.1%), they still remain low when compared to HICs such as South Korea, Canada, and Iceland, where almost 70% of the hypertensive patients were accurately diagnosed [50, 52]. A wide variability was observed in the proportion of patients undergoing treatment (27.9%–78.9%) [10, 17, 25, 27, 29, 30]. Similar to the observations made on awareness, the participants who received treatment mainly belonged to geriatric, female, those with comorbidities and educated cohorts [10, 24, 25]. Although treatment rates in Saudi Arabia are higher, compared with UAE and Iran (45% and 43%, respectively), the treatment rates in HICs were reported to be > 70% [25, 52]. Further, proportion of patients adhering to antihypertensive medications was reported to be low in Saudi Arabia (22.5%) [28], which was similar to the findings from other Middle Eastern countries like OPT (16.9%) [53], but was lower than UAE (42.0%) [54]. Consistent with these findings, previously published studies have reported higher treatment (74.0%) and lower adherence rates (6.2%) within Saudi Arabia [52, 55]. Likewise, a huge variation was observed in the proportion of patients achieving BP control (27.0%–45.0%) [10, 17, 25, 29, 30], while HICs including Canada and Germany reported higher BP control (50.0%–69.0%) [52].

**Fig. 3:**
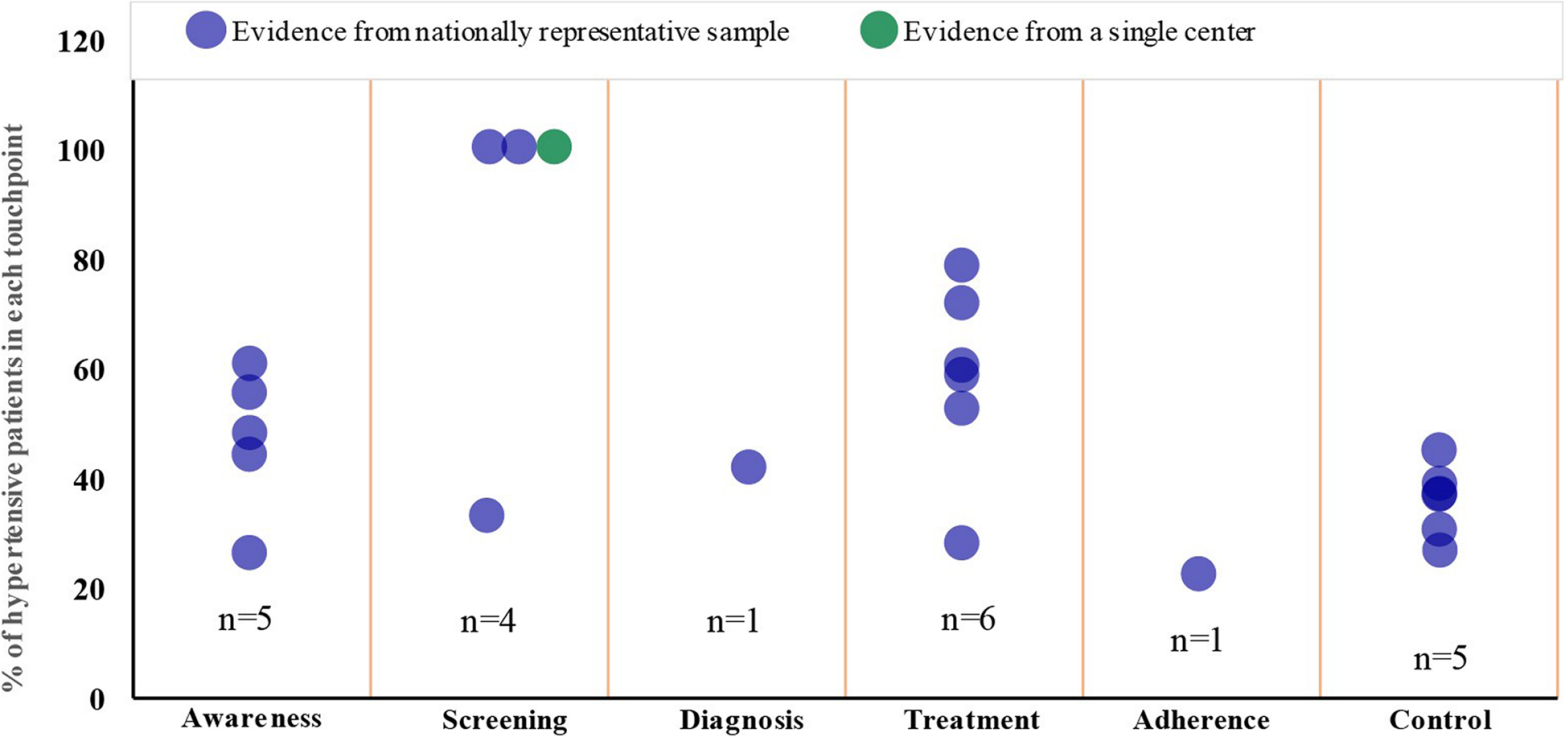
Dot plot showing evidence gap map presenting number of sources (n) stratified per patient journey touchpoints for hypertension

Overall, when compared with other Middle Eastern countries, the prevalence of hypertension reported within community-settings in Saudi Arabia (14.0%–36.0%) was found to be comparable with countries like Iran, Turkey, and Oman (19.2%–33.7%) [56]. The awareness in UAE, Iran and OPT ranged from 45.0%–57.0%, which was higher compared with Saudi Arabia (27.6%–61.1%). Treatment (27.9%–78.9%) and control rates (27.0%–45.0%) within Saudi Arabia were comparatively higher than that in Iran (treatment; 43.0% and control; 16.0%) and UAE (treatment; 45.0% and control; 14.0%), respectively [25]. The overall increasing trends in hypertension seen across all the patient journey touchpoints pose a major challenge to the healthcare system in Saudi Arabia. The gender differences observed in hypertension prevalence has a physiological basis as suggested by few gender-based analytical studies [57, 57–59]. The experimental models of hypertension show a higher level of angiotensin II receptors (type 1; AT_1_), which are mainly responsible for vasoconstriction, sodium reabsorption, and eventual rise in BP, in males vs. females [59]. Additionally, other challenges include disparity across various sections of the community with regards to the knowledge and attitude towards preventive healthcare, unhealthy lifestyle behavior, and lack of motivation among Saudi people to self-manage hypertension [10, 17]. Moreover, low numbers of evidence pertaining to each critical patient journey touchpoint can impede adequate implementation of health interventions among hypertensive patients (Fig. 3).

### Dyslipidemia: gaps along patient journey touchpoints

The prevalence of dyslipidemia has increased steeply over the last many years from 12.5% to 43.0% [11, 29, 41, 43]. This increase could be attributed to lifestyle changes owing to rapid urbanization, poor diet, and low physical activity [35]. Dyslipidemia (with increased low-density lipoprotein cholesterol [LDL–C]) was the leading CV risk factor (32.1%–68.6%) among other risk factors, including hypertension, diabetes, smoking, obesity (body mass index [BMI] ≥ 30 kg/m^2^), or abdominal obesity [24, 40, 41]. These findings were comparable to HICs in Western Europe (40.0%–60.0%) and North America (40.0%–45.0%) [60]. Elevated LDL-C over a prolonged period can increase the risk of atherosclerotic cardiovascular diseases (ASCVDs) events by almost five times, which reiterates the importance of maintaining an optimum lipid profile [61]. Dyslipidemia was also positively associated (62.0%) in patients with increased body mass index (BMI) and waist circumference (WC) along with faulty lifestyle and dietary habits such as inadequate sleep, caffeine intake, and consumption of sugary carbonated beverages [41].

A wide variability in dyslipidemia awareness level (10.5%–47.3%) was observed in geriatric patients, uneducated individuals, and rural dwellers in Saudi Arabia [33, 37]. The awareness rates pertaining to CVD risk factors including dyslipidemia in the urban educated population in Saudi Arabia were also within this range (47.1%) [62]. A small proportion of patients (34.6%) visiting the PHC underwent lipid screening in Saudi Arabia. This could be because of lack of awareness pertaining to the importance of regular screenings, especially among younger individuals (> 20 years), gender disparity, and unemployment [38]. On the other hand, UAE reported higher screening rates (49.3%) in > 8000 patients [63].

As reported by CEPHEUS study, a high proportion of patients (94.0%) with high and very high ASCVD risk underwent statin therapy. This reinstates the importance of dyslipidemia management in the primary prevention of CVD events, especially in individuals with multiple CVD risk factors [36]. However, a lower proportion of patients (40.6%) were receiving lipid-lowering treatment for secondary prevention of CVDs. This was due to the inequality in wealth distribution persistent in Saudi Arabia that led to inequality in accessing adequate treatment and poor prognosis of CVDs. These observations agree with previous Saudi studies that reported underwhelming use of high-intensity statins for secondary prevention in patients with ASCVD or sub-optimal adherence to prevention guidelines in secondary prevention of coronary events [64, 65]. Conversely, this trend was not observed in other HICs such as UAE, Canada, and Sweden, where more than half of the population received lipid-lowering treatment for secondary prevention [39]. Furthermore, a higher adherence rate (62.0%–77.4%) was observed in patients with type 2 DM and dyslipidemia which was the result of extensive dyslipidemia education programs that were undertaken at the beginning of the study [42]. The anecdotal evidence for adherence (45.0%) further calls for urgent implementation of such education programs across various communities in Saudi Arabia. Notably, the control rates were found to be lower (23.0%–41.5%) among the patients, which could be due to non-indulgence in physical activity or not having a controlled diet which reemphasizes the important role of non-pharmacological therapy along with drug therapy [36, 40]. Moreover, drug intolerance, inadequate PHC follow-up and using medications that are not evidence-based, especially in females, can hamper optimal lipid control [36].

While, the prevalence of dyslipidemia in Northern Emirates was reported to be slightly higher (72.5%) [66] compared to Saudi Arabia (12.5%–62.0%), the Gulf Registry of Acute Coronary Events (Gulf RACE) encompassing countries such as Bahrain, Kuwait, Qatar, Oman, UAE, and Yemen had a lower prevalence (32.7%) [67]. Egypt reported lower screening rate (8.6%) compared to Saudi Arabia (34.6%). In other middle-eastern countries like Jordan, Lebanon, UAE, Kuwait, and Oman, higher treatment (55.9%) and control rates (52.4%) were observed, compared to that in Saudi Arabia (treatment rate: 40.0% and control rate: 28.0%–41.5%, respectively) [36, 38, 42].

**Fig. 4:**
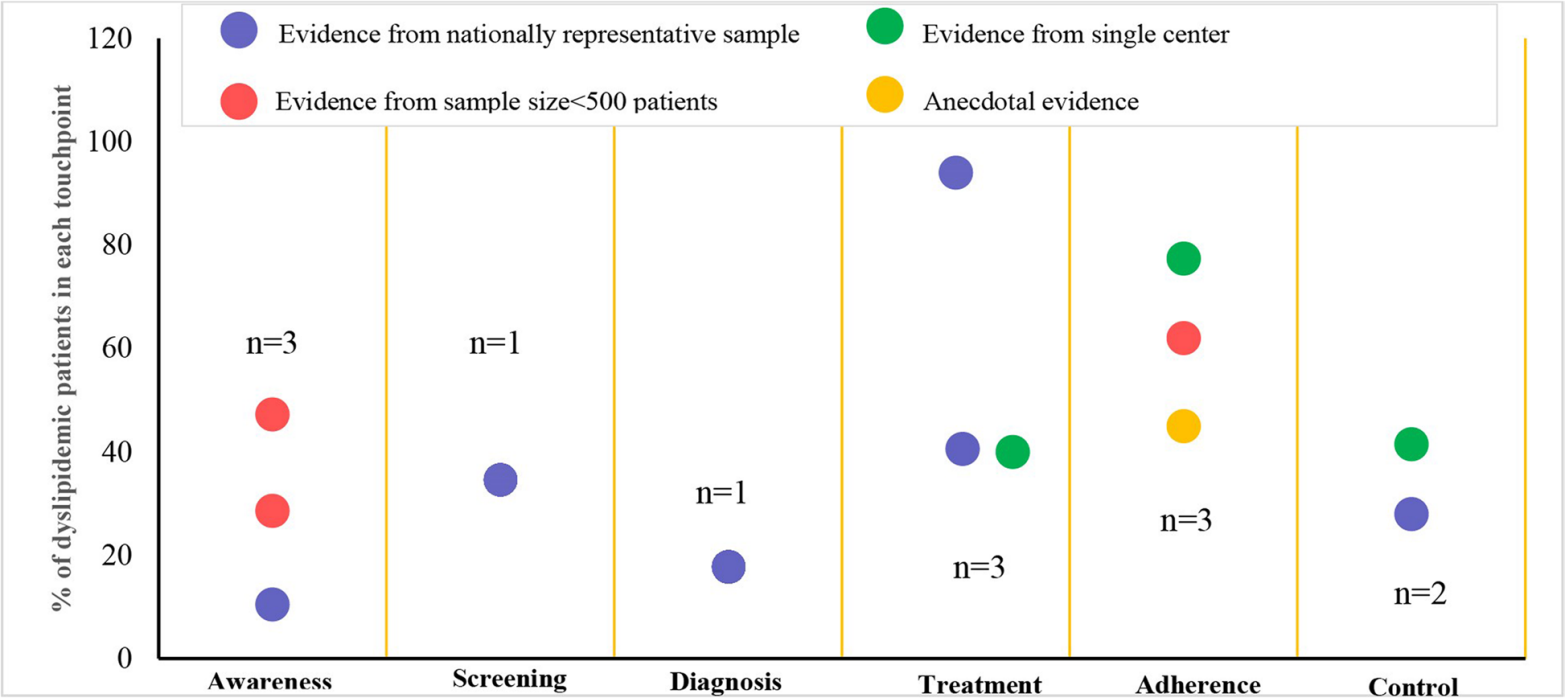
Dot plot showing evidence gap map presenting number of sources (n) stratified per patient journey touchpoint for dyslipidemia

Overall, a huge scarcity in data for dyslipidemia along each patient journey touchpoint has been observed in Saudi Arabia that deters targeted management strategies (Fig. 4).

### Scope of recommendations for enhanced patient care and health outcomes

The Saudi Arabia healthcare system has rapidly evolved over the last 20 years and is home to world-class hospitals and efficient healthcare facilities. The Saudi Healthcare National Transformation Program (NTP) has been a driving force for Saudi Vision 2030 to bring about a transformation in the healthcare sector by prioritizing NCD prevention [29, 68]. However, despite the availability of adequate resources and implementation of multiple initiatives, numerous evidence gaps along each patient journey touchpoint in hypertension and dyslipidemia still persist. This may be partly due to less NCD research output from Saudi Arabia compared with other Middle Eastern countries and a smaller number of national awareness programs and multi–faceted healthcare policies [29, 68]. Furthermore, the current COVID-19 pandemic has adversely impacted the overall management of hypertension and dyslipidemia owing to partial and complete lockdowns and cancellation of in–person visits to PHCCs [69].

As the future steps to reduce CVD burden within the Saudi population, active collaborations are needed between patients, HCPs, community/hospital pharmacists, and healthcare policy makers to enhance coordination between primary care and health services delivery systems, backed by adequate funding and leadership for uniform implementation [70]. Such collaborations can also help identify barriers along the patient journey and plan future research to address them. This could be accomplished by emphasizing national-level epidemiological studies over region-based data, with bias-free study designs and protocols for hypothesis testing. Further, dissemination of study results adhering to standardized reporting guidelines such as STrengthening the Reporting of OBservational studies in Epidemiology (STROBE) can ensure study ethics and methodological robustness [71].

Table 4 represents recommendations for enhanced patient care and health outcomes across all patient journey touchpoints, designed by all authors through mutual consensus.

**Table 4:** Recommendations for enhanced patient care and health outcomes

Prevalence	Recommendations
	***Patients*** • Adopting healthy lifestyle modifications • Reducing salt intake • Increasing physical activity ***HCPs*** • Collecting RWD using EMR to further strengthen epidemiological research over time and across regions [72] ***Healthcare policy makers*** • Conducting public health programs that emphasizes lifestyle modification • Addressing gender inequalities by improving access to safe and culturally acceptable exercise facilities for women [73] • Levying higher taxation on tobacco products/sugar based carbonated drinks [24] • Establishing rehabilitation centers for smokers [4] • Promoting preventive attitude through regular campaigns at public places, schools, and social media [74]
***Patient journey touchpoints***	
**Awareness**	***Patients*** • Regularly participating in community-awareness programs • Encouraging adolescents and teenagers in their families and neighborhood to adopt healthy lifestyle and early screening for timely diagnosis and management ***HCPs*** • Imparting knowledge regarding symptoms, risk factors, causes and complications with use of: ▪ Simple lay languages and posters with easy takeaway messages, e.g., ‘know your numbers’ (in context of hypertension) ▪ Graphical representation, or handouts to explain the meaning of cholesterol and heart diseases ▪ Internet-based education and audiovisual aids • Organizing CME programs highlighting: ▪ Advanced therapies ▪ Updated treatment guidelines ▪ New risk assessment tools validated in local population [12] ▪ Training on the integrated management of hypertensive patients with comorbid conditions [75] ▪ CV risk factor management using advanced technologies [12] ***Healthcare policy makers*** • Development of health-related mobile applications that has features such as [76] ▪ Combined intuitive interfaces and appealing design ▪ User-friendly features (with continued feedback and textual explanation) ▪ Timely reminders ▪ Disease-specific educational content • Educating youth to debunk myth that lifestyle changes are applicable only to elderly or patients • Increasing accessibility to healthcare facilities, especially among the geriatric population by increasing: ▪ Public transport ▪ Number of ramps and handrails [15]
**Screening and Diagnosis**	***Patients*** • Undergoing screening at PHCCs rather than community screening [38] ***HCPs*** • Promoting screening at PHCCs for following patient subsets [77]: ▪ Periodical screening of lipid profile for all individuals ≥ 40 years or with ≥ one risk factor [35] ▪ Universal screening for general population [77] ▪ Selective screening for individuals admitted to acute coronary units or history of MI [77] ▪ Family cascade screening for patients with FH [77] • Clearly defining the cut-off values for the clinical parameters depending on age, gender, and presence of CVD risk factors for accurate diagnosis ***Healthcare policy makers*** • Imparting knowledge through health promotion campaigns regarding the importance of early screening [32]
**Treatment**	***Patients*** • Constant collaboration with HCPs for: ▪ Better disease understanding ▪ Better treatment decisions ▪ Better accountability in disease management and control ***HCPs*** • Considering factors such as easy availability (easy to refill), easy to take (once a day single pill combination), along with a reminder methodology while prescribing drugs • Using multifaceted approach involving systematic use of diagnostic tools while deciding on the mono- or dual-therapy approach in early stages [37] • Using of simplified treatment regimen and combination pill concept ***Healthcare policy makers*** • Involving a team including clinical pharmacists and nurses in patient management [78]
**Adherence and Control**	***Patients*** • Involving family members for seeking medication reminders assistance at home • Being proactive for self-management of health (e.g., self-monitoring of BP) • Using technologies, e.g., iPhone users to set medication reminders record [79] ***HCPs*** • Imparting constant feedback to patients’ regarding their health status • Using self-reported questionnaire pertaining to hypertension-related complications to promote self-management attitude ***Healthcare policy makers*** • Imparting multidisciplinary educational program addressing misinformation on AEs of the drugs [42]

*Abbreviations: AE* adverse event, *BP* blood pressure, *CME* continuing medical education, *CV* cardiovascular, *CVD* cardiovascular diseases, *EMR* electronic medical records, *FH* familial hypercholesterolemia, *HCPs* healthcare practitioners, *MI* myocardial infarction, *PHCCs* primary healthcare clinics, *RWD* real-world data

### Limitations

This review had several limitations. Firstly, we excluded studies conducted in specific patient subgroups (pregnant women, patients with co-morbidities). However, the reason for this exclusion was solely based on the objective to understand and highlight the evidence gaps in the patient journey of hypertension and dyslipidemia, respectively. Furthermore, adding data from patients with comorbidities would have increased literature volume diluting the objectives of this study or skew the overall effect size resulting from additional treatment/patient arms. As a result, the gaps in hypertension and dyslipidemia patients’ journey may not be extrapolated from participants with comorbidities [80]. Nevertheless, this could have led to the underestimation of the overall data and can be a perspective for future research by designing appropriate study methodology. Secondly, we chose to exclude non-English language studies to overcome the reduced traceability of studies that are published in local language and the associated translation costs, although attempts were made to include local language studies with data validated by local experts [81]. Thirdly, we excluded specific study types (i.e., thesis abstracts; letters to the editor, and editorials) to ensure that studies of high quality and strong recommendations (systematic reviews followed by observational studies and narrative reviews) be considered to enable the readers make evidence-informed decisions [76]. Furthermore, we did not include publication bias analysis for the selected studies, and we did not calculate the pooled estimates for each touchpoint owing to the wide variability in data. We considered only TC while including studies for dyslipidemia without considering additional variables such as LDL-C, triglycerides (TG), and high-density lipoprotein cholesterol (HDL-C). Finally, we could not include information on the efficacy of specific treatment intervention(s). This was again attributed to our underlying objective for this study which was to only highlight the evidence gaps in the patient journey.

## Conclusion

This review presented detailed insights across various patient journey touchpoints during the management of hypertension and dyslipidemia in Saudi Arabia. A comprehensive search strategy was applied, and major data gaps were identified. Addressing these data gaps can aid the healthcare stakeholders to strategize a goal-oriented approach for achieving positive CV outcomes among patients and further strengthen the Saudi Arabia healthcare system. Multi-faceted healthcare policies including well-defined protocols for NCD prevention along with collective effort from the triad of patients, HCPs, and the healthcare policy makers can amplify the quality of care and better utilization of healthcare resources. Furthermore, this review emphasizes the need for more epidemiological research, national-level surveillance data highlighting the changing patterns of CVD risk factors, and mortality among key subpopulations including high-risk individuals, women, and children.

## Supplementary Information

**Additional file 1.** Definitions for each patient journey touchpoints for hypertension and dyslipidemia.

**Additional file 2.** Complete search strategy with keywords and Boolean operators along with inclusion and exclusion criteria for hypertension and dyslipidemia.

## Data Availability

All data generated or analyzed during this study are included in this published article (and its supplementary information files). Anecdotal data as personal communication from the local expert in the context of dyslipidemia that has been used in this study will be available on request from the corresponding author.

## Abbreviations

ACE study: Africa Middle East Cardiovascular Epidemiological study
ASCVD: Atherosclerotic cardiovascular diseases
AT1: Angiotensin II receptor type 1
BMI: Body mass index
BP: Blood pressure
CAD: Coronary artery diseases
CEPHEUS study: Centralized Pan-Middle East Survey on the under-treatment of hypercholesterolemia (CEPHEUS) study
COVID-19: Coronavirus disease
CV: Cardiovascular
CVDs: Cardiovascular diseases
DBP: Diastolic blood pressure
DM: Diabetes mellitus
HCPs: Healthcare practitioners
HDL-C: High-density lipoprotein cholesterol
HIC: High income countries
IPD: Incidence and Prevalence Database
KSA WHS: Kingdom of Saudi Arabia World Health Survey
LDL–C: Low-density lipoprotein cholesterol
MAPS: Mapping the Patient Journey Towards Actionable Beyond the Pill Solutions
MENA: Middle East, North Africa
MMAS: Morisky Medication Adherence Scale
NCDs: Non communicable diseases
NTP: National Transformation Program
OPT: Occupied Palestinian Territory
PHC: Primary healthcare
PHCCs: Primary healthcare clinics
PRISMA: Preferred Reporting Items for Systematic Reviews and Meta-Analyses
PURE study: Prospective Urban Rural Epidemiology study
SBP: Systolic blood pressure
SHIS: Saudi Health Interview Survey
T2D: Type 2 diabetes
TC: Total cholesterol
TG: Triglycerides
UAE: United Arab Emirates
WC: Waist circumference
WHO: World Health Organization
