# Supplementary material for: Patient journey for hypertension and dyslipidemia in Saudi Arabia: highlighting the evidence gaps
Source: Arch Public Health. 2023 Jul 3;81:122. doi: 10.1186/s13690-023-01121-3 (PMC10316580; doi:10.1186/s13690-023-01121-3)
Supplement: Supplementary file 1 — Additional file 1. Definitions for each patient journey touchpoints for hypertension and dyslipidemia. [file 13690_2023_1121_MOESM1_ESM.docx]

**Table 1A**: Definitions for each patient journey touchpoints for hypertension

| **Patient journey touchpoints** | **Definitions** |
| --- | --- |
| Awareness | Self-reported or any prior diagnosis of hypertension by a healthcare professional |
| Screening | Proportion of respondents who had their blood pressure (BP) measured by a doctor or any other health worker |
| Diagnosis | Patients diagnosed with hypertension by a healthcare professional |
| Treatment | Use of a hypertension medication for management of the respondent’s high BP |
| Adherence | Proportion of respondents indicating adherence and/or compliance to the prescribed BP medications |
| Control | Proportion of patients achieving a target BP of ≤140/90 mmHg with treatment |

**Table 1B**: Definitions for each patient journey touchpoints for dyslipidemia

| **Patient journey touchpoints** | **Definitions** |
| --- | --- |
| Awareness | Self-reported or any prior diagnosis of high total serum cholesterol by a healthcare professional |
| Screening | Proportion of respondents who had their cholesterol levels measured by a doctor or any other health worker |
| Diagnosis | Patients diagnosed with dyslipidemia disorder by a healthcare professional |
| Treatment | Use of medications for management of the respondent’s high cholesterol |
| Adherence | Proportion of respondents indicating adherence and/or compliance to the prescribed cholesterol lowering medications |
| Control | Proportion of patients achieving a target cholesterol of ≤5.0 mmol/L OR ≤200 mg/dL with treatment |
