# Supplementary material for: Patient journey for hypertension and dyslipidemia in Saudi Arabia: highlighting the evidence gaps
Source: Arch Public Health. 2023 Jul 3;81:122. doi: 10.1186/s13690-023-01121-3 (PMC10316580; doi:10.1186/s13690-023-01121-3)
Supplement: Supplementary file 2 — Additional file 2. Complete search strategy with keywords and Boolean operators along with inclusion and exclusion criteria for hypertension and dyslipidemia. [file 13690_2023_1121_MOESM2_ESM.docx]

**Supplementary Table 2A. Strategy for Structured Literature Search on Hypertension**

| **Hypertension** | | |
| --- | --- | --- |
| **Search String** | **Inclusion Criteria** | **Exclusion Criteria** |
| hypertension OR blood pressure OR hypertensives  AND  epidemiology OR prevalence OR incidence OR national OR survey OR registry  AND  awareness OR knowledge OR health literacy OR screening diagnosis OR diagnosed OR undiagnosed OR treatment OR treated OR untreated OR control OR controlled OR uncontrolled OR adherence OR compliance OR adhere OR therapy OR non-adherence  AND  Saudi Arabia | **Time period**: January 1, 2010 –December 31, 2021  **Language**: English  **Species**: Humans, Human  Hypertension relevant patient journey data available in Kingdom of Saudi Arabia | <18 years of age  Not hypertension  Relevant patient journey data NA  Full text NA  Specific patient subgroups such as patients with comorbidities, pregnant women  Not English language  Case studies, letter to editors, editorials  Duplicate records  Data lacking national representativeness  Data not from representative country |
| **Abbreviations:** NA, not available | | |

**Supplementary Table 2B. Strategy for Structured Literature Search on Dyslipidemia**

| **Dyslipidemia** | | |
| --- | --- | --- |
| **Search String** | **Inclusion Criteria** | **Exclusion Criteria** |
| dyslipidemia OR hypercholesterolemia OR cholesterol OR triglycerides OR LDL  AND  epidemiology OR prevalence OR incidence OR national OR survey OR registry OR Statistics  AND  health literacy OR screening OR awareness OR knowledge OR treated OR treatment OR diagnosis OR undiagnosed OR diagnosed OR therapy OR controlled OR control OR uncontrolled OR adherence OR adhere OR compliance  AND  United Arab Emirates OR Algeria OR South Africa OR Africa OR Egypt OR Saudi Arabia OR Middle East | **Time period**: January 1, 2010 –December 31, 2021  **Language**: English  **Species**: Humans, Human,  Dyslipidemia relevant patient journey data available in Kingdom of Saudi Arabia | <18 years of age  Not dyslipidemia  Relevant patient journey data NA  Full text NA  Specific patient subgroups such as patients with comorbidities, pregnant women  Not English language  Case studies, letter to editors, editorials  Duplicate records  Data lacking national representativeness  Data not from representative country |
| **Abbreviations:** LDL, low-density lipoprotein NA, not available | | |
